# The Impact of Liver Cirrhosis on Clinical Outcomes in Patients With Acute Pancreatitis: A Systematic Review and Meta-Analysis

**DOI:** 10.7759/cureus.84567

**Published:** 2025-05-21

**Authors:** Helai Hussaini, Rahman Hameed Mohammed Abdul, Mohammed Qasim Rauf, Renad Fahim, Olaniyi Fadeyi, Sandipkumar S Chaudhari, Ihtisham Habib, Danish Allahwala

**Affiliations:** 1 Internal Medicine, West Anaheim Medical Center, Anaheim, USA; 2 Gastroenterology and Hepatology, Royal Derby Hospital, Stoke-on-Trent, GBR; 3 Department of Orthopaedic Surgery, Hillingdon Hospital, London, GBR; 4 Medicine, Jinnah Sindh Medical University, Karachi, PAK; 5 Cardiothoracic Surgery, University of Alabama at Birmingham, Birmingham, USA; 6 Family Medicine, University of North Dakota School of Medicine and Health Sciences, Fargo, USA; 7 Internal Medicine, Medical Teaching Institute, Lady Reading Hospital, Peshawar, PAK; 8 Nephrology, Fatima Memorial Hospital, Karachi, PAK

**Keywords:** acute pancreatitis, disease severity, liver cirrhosis, meta-analysis, mortality

## Abstract

This systematic review and meta-analysis analyzed the impact of liver cirrhosis (LC) on clinical outcomes in patients hospitalized with acute pancreatitis (AP). We searched multiple databases, including PubMed, Embase, Web of Science, and the Cochrane Library from inception to April 26, 2025, and included studies comparing outcomes between AP patients with and without LC. From the 1,233 initially identified studies, eight retrospective studies with a pooled sample of 3,513,655 patients were included in the final analysis, with an LC prevalence of 2.90%. Meta-analysis revealed that AP patients with LC had significantly higher mortality risk compared to non-cirrhotic patients [risk ratio (RR): 2.09, 95% confidence interval (CI): 1.74-2.52], with considerable heterogeneity (I² = 66%). Subgroup analysis of studies reporting adjusted effect estimates confirmed this finding (RR: 1.94, 95% CI: 1.52-2.48) with lower heterogeneity (I² = 28%). Cirrhotic patients also experienced significantly more severe AP (RR: 2.79, 95% CI: 1.53-5.11) with no heterogeneity among studies (I² = 0%). However, analysis showed no significant difference in acute kidney injury (AKI) risk between cirrhotic and non-cirrhotic patients (RR: 1.09, 95% CI: 0.84-1.41).

Our findings highlight the importance of early identification and vigilant monitoring of cirrhotic patients with AP, who may benefit from coordinated multidisciplinary care involving hepatologists, nephrologists, and infectious disease specialists. The limitations of the study include the retrospective design of all included studies, insufficient granularity for comprehensive subgroup analyses based on liver disease etiology or fibrosis stage, and inconsistent reporting of key clinical outcomes across studies. Future prospective research should address these limitations to better inform personalized management strategies.

## Introduction and background

Acute pancreatitis (AP) is a sudden inflammatory condition of the pancreas with variable clinical presentations ranging from mild, self-limiting disease to severe, life-threatening illness characterized by systemic inflammatory response, multiorgan failure, and high mortality [1-2]. It remains one of the most common gastrointestinal causes of hospital admissions worldwide, with increasing incidence noted in both developed and developing countries [3]. The etiology of AP is multifactorial, with gallstones and alcohol use being the leading contributors. Despite advancements in supportive care, the burden of complications and mortality associated with severe AP remains substantial [4]. 

Liver cirrhosis (LC), the end-stage of chronic liver disease (CLD), is characterized by progressive fibrosis, portal hypertension, and impaired liver function [5]. Globally, cirrhosis contributes significantly to morbidity and mortality, particularly in populations with high prevalence of hepatitis, alcohol abuse, or non-alcoholic fatty liver disease [6]. Patients with cirrhosis are known to have immune dysfunction, altered coagulation, and compromised organ perfusion, which can influence outcomes when faced with acute systemic illnesses [7]. When AP occurs in patients with underlying LC, the interplay between the two major organ failures raises concern for compounded clinical outcomes [8]. 

Emerging evidence suggests that cirrhosis may exacerbate the course of AP through multiple mechanisms, including reduced hepatic clearance of inflammatory mediators, impaired host defense, increased risk of infections, and susceptibility to renal and cardiovascular complications [9]. In addition, cirrhosis-associated portal hypertension and ascites may complicate the management of fluid resuscitation in AP, further challenging the clinical course [10]. However, the extent to which LC contributes to worse outcomes in patients with AP remains variably reported across the literature. 

Given the increasing prevalence of both AP and LC globally and the clinical importance of risk stratification in guiding management decisions, a comprehensive synthesis of available evidence is needed. Therefore, we conducted a systematic review and meta-analysis to evaluate the impact of LC on clinical outcomes in patients hospitalized with AP. The primary aim was to assess whether LC is associated with a higher risk of mortality, severity, and acute kidney injury (AKI). Our findings aim to inform clinical decision-making, guide risk assessment, and highlight areas requiring further research in this complex patient population.

## Review

Methodology 

Eligibility Criteria 

Studies were eligible for inclusion if they involved adult patients diagnosed with AP and compared outcomes between those with LC and those without any documented history or clinical evidence of cirrhosis. The main outcome of interest was mortality, while secondary outcomes included the severity of AP and the occurrence of AKI. Eligible study designs encompassed randomized controlled trials, as well as prospective and retrospective cohort studies. Case reports and case series were excluded from the analysis. 

Information Sources and Search Strategy 

A comprehensive literature search was conducted to identify relevant studies examining the impact of LC on outcomes in patients with AP. We searched PubMed, Embase, Web of Science, and the Cochrane Library from their inception to April 26, 2025, using a combination of Medical Subject Headings (MeSH) terms and free-text keywords related to “acute pancreatitis,” “liver cirrhosis,” and “clinical outcomes.” The search strategy was tailored to each database and included Boolean operators to enhance sensitivity. Reference lists of all included studies and relevant reviews were also screened manually to identify any additional eligible articles. No language restrictions were applied during the search process. 

Selection Process 

All records identified through the database searches were imported into reference management software, and duplicates were removed. Two independent reviewers then screened the titles and abstracts to assess eligibility based on the predefined inclusion and exclusion criteria. Studies deemed potentially relevant were retrieved in full text and assessed independently by the same reviewers for final inclusion. Any disagreements during the selection process were resolved through discussion or consultation with a third reviewer. The entire selection process was documented using the PRISMA (Preferred Reporting Items for Systematic Reviews and Meta-Analyses) flow diagram. 

Data Extraction 

Data extraction was performed independently by two reviewers using a standardized data collection form developed before the review. For each included study, the reviewers extracted information on study characteristics (such as author, publication year, country, and study design), patient demographics, diagnostic criteria for AP and LC, sample size, and reported outcomes. Outcomes of interest included mortality, severity of AP, and incidence of AKI. Where relevant data were missing or unclear, attempts were made to contact the study authors for clarification. Discrepancies between reviewers were resolved through discussion or consultation with a third reviewer to ensure accuracy and consistency. 

Data Analysis 

We synthesized data from the selected studies by calculating risk ratios (RRs) along with their corresponding 95% confidence intervals (CIs) to evaluate the association between cirrhosis and the outcomes of interest. When studies provided adjusted effect estimates, these were utilized directly. In instances where adjusted measures were not available, unadjusted RRs were computed using the extracted raw data. 

To assess the degree of variability among the included studies, we employed the I² statistic, which quantifies the proportion of total variation attributable to heterogeneity rather than chance. Given the anticipated clinical and methodological differences across studies, a random-effects model was adopted for the meta-analysis to account for potential between-study heterogeneity. This approach assumes that the true effects vary across studies and provides a more conservative estimate of the overall effect size. Statistical significance was determined by examining whether the 95% CI for the pooled RR excluded the null value. All analyses were conducted using Review Manager (RevMan) software, version 5.4.1. 

Results 

**Figure 1:**
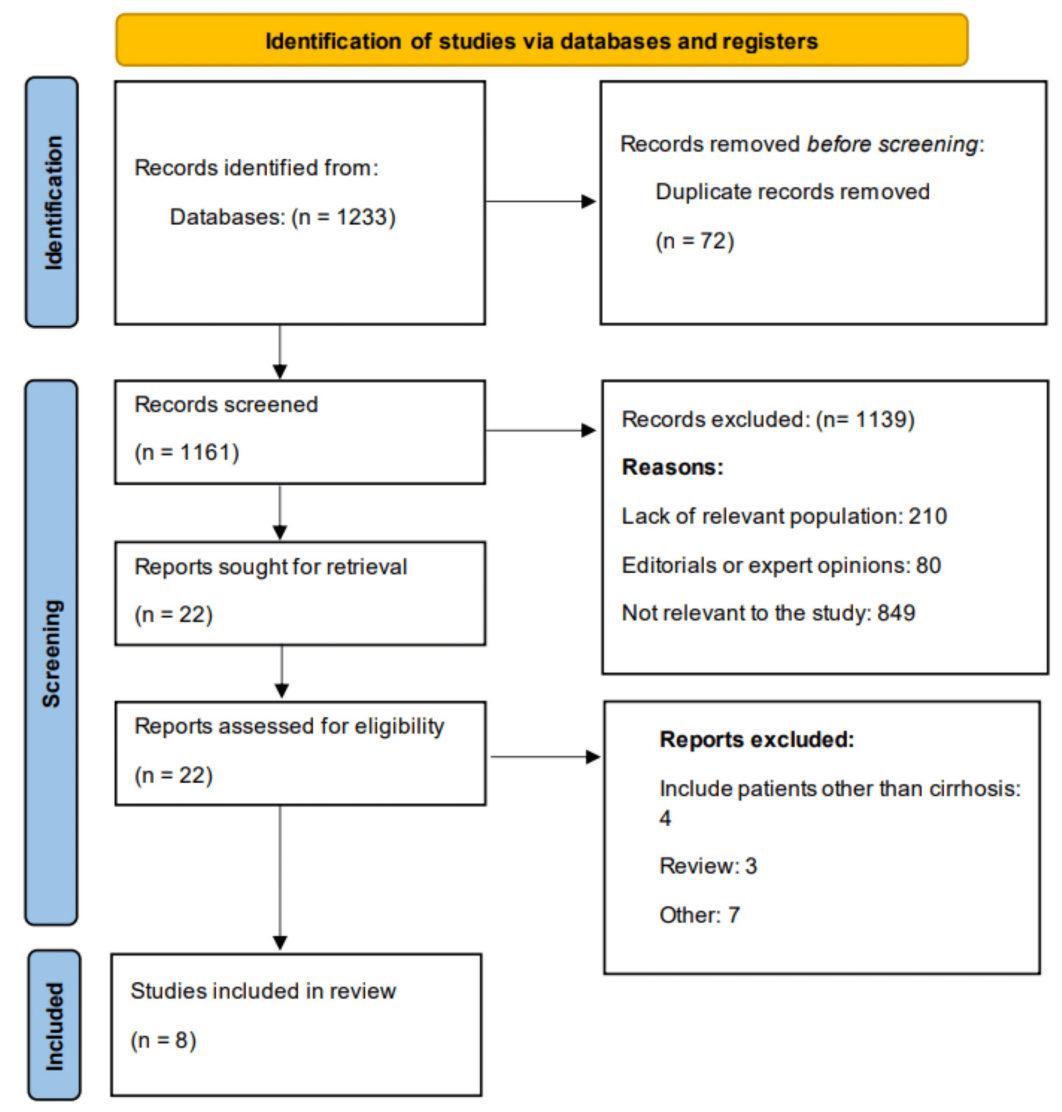
PRISMA flowchart showing study selection process PRISMA: Preferred Reporting Items for Systematic Reviews and Meta-Analyses

Overall, 1,233 studies were identified through online database searching. After removing duplicates, followed by title and abstract screening, 22 studies underwent full-text screening. Finally, eight studies were included in the meta-analysis. Figure 1 shows the PRISMA flowchart of study selection. Table 1 shows characteristics of the included studies. The pooled sample size was 3,513,655. Pooled prevalence of LC was 2.90%. All studies were retrospective. Four studies were conducted in the United States, two in Korea, and one each in Germany and China. Table 2 presents the quality assessment of the included studies.

**Table 1:** Characteristics of the included studies BMI: body mass index; LC: liver cirrhosis; NR: not reported

Authors	Year	Design	Country	Sample size		Male	Age	Diabetes	Hypertension	BMI
				LC	Non-LC					
Abushamma et al. [11]	2018	Retrospective	United States	40	80	60	60.4	NR	NR	26.7
Aziz et al. [12]	2025	Retrospective	United States	14,180	723,959	402,895	50.9	199,353	NR	NR
Jang et al. [13]	2021	Retrospective	Korea	32	210	215	47	38	69	21.9
Liu et al. [14]	2022	Retrospective	China	46	585	348	60.38	167	NR	NR
Mahfouz et al. [15]	2022	Retrospective	United States	7,587	66,508	44,987	NR	NR	NR	NR
Park et al. [16]	2019	Retrospective	Korea	63	609	446	50.91	136	155	NR
Simons-Linares et al. [17]	2020	Retrospective	United States	80,093	2,721,600	1,445,640	52.5	NR	NR	NR
Vagel et al. [18]	2022	Retrospective	Germany	52	104	112	49.36	NR	NR	24.76

**Table 2:** Quality assessment of included studies using the Newcastle-Ottawa Scale

Authors	Selection (0 to 4)	Comparability (0 to 2)	Outcome (0 to 3)	Grade
Abushamma et al. [11]	3	2	2	Good
Aziz et al. [12]	4	2	2	Good
Jang et al. [13]	3	2	2	Good
Liu et al. [14]	4	2	2	Good
Mahfouz et al. [15]	3	1	2	Fair
Park et al. [16]	3	1	2	Fair
Simons-Linares et al. [17]	4	2	3	Good
Vagel et al. [18]	3	1	3	Good

Meta-Analysis: Impact of LC on AP Outcomes 

**Figure 2:**
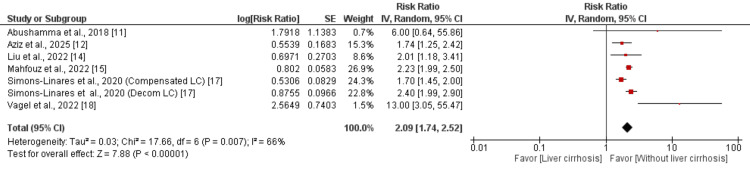
Forest plot comparing mortality References [11-12,14-15,17-18] CI: confidence interval

Mortality in AP with LC: The meta-analysis of six studies (with one study included twice for separate analysis of compensated and decompensated cirrhosis) demonstrates significantly higher mortality risk in AP patients with LC. Patients with LC had more than twice the mortality risk compared to non-cirrhotic patients (RR: 2.09, 95% CI: 1.74 to 2.52) as shown in Figure 2. However, considerable heterogeneity was observed among the included studies (I² = 66%), suggesting variability in the reported effect sizes. 

We performed subgroup analysis to evaluate the effect of LC on mortality in AP patients, focusing exclusively on studies reporting adjusted effect estimates. The pooled analysis of these methodologically robust studies demonstrated that patients with LC had a significantly higher risk of mortality compared to non-cirrhotic patients (RR: 1.94, 95% CI: 1.52 to 2.48). Importantly, this analysis showed low heterogeneity among the included studies (I² = 28%).

**Figure 3:**
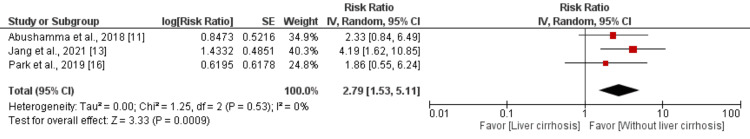
Forest plot comparing severity of AP References [11,13,16] AP: acute pancreatitis; CI: confidence interval

Severity of AP with LC: Analysis of three studies examining AP severity revealed that LC patients experience significantly more severe AP compared to non-cirrhotic patients (RR: 2.79, 95% CI: 1.53 to 5.11) as shown in Figure 3. Notably, there was no heterogeneity among these studies (I² = 0%), indicating consistent findings across all included research. 

**Figure 4:**
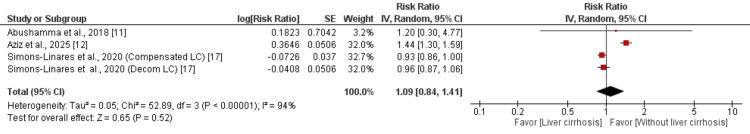
Forest plot comparing AKI References [11-12,17] AKI: acute kidney injury; CI: confidence interval

AKI risk in cirrhotic AP: Regarding AKI, analysis of three studies (with one study counted twice for separate analysis of compensated and decompensated cirrhosis) showed no significant difference in AKI risk between cirrhotic and non-cirrhotic AP patients (RR: 1.09, 95% CI: 0.84 to 1.41)as shown in Figure 4. Substantial heterogeneity was present among these studies (I² = 94%), indicating considerable variability in the reported outcomes. 

Discussion 

This meta-analysis assessed the effect of LC on the outcomes among patients with AP. This study showed that LC increases the severity of AP and mortality. Previous meta-analysis also showed increased risk of death, severity, systemic and local complications were higher in the CLD subjects [19]. However, our study is different from this as we focused only on LC subjects and included recently conducted studies. 

AP ranks among the leading gastrointestinal conditions necessitating hospitalization. The incidence of AP has been on the rise, a trend attributed to contemporary lifestyle factors such as increased alcohol consumption, diets rich in fats, and a growing prevalence of obesity and gallstone disease [20]. The primary causes of AP include gallstones, responsible for approximately 40-70% of cases, and chronic alcohol use, accounting for 17-25% of cases [21]. Hypertriglyceridemia is another notable cause, implicated in up to 22% of cases. The remaining instances are due to a variety of less common factors, including certain medications, infections, and autoimmune conditions [22]. 

Recognizing and managing patients with underlying LC is crucial, as it can significantly impact the clinical course of AP. In cases of severe AP, the inflammatory response leads to increased vascular permeability, causing fluid to shift from the bloodstream into surrounding tissues - a process known as third spacing. This results in significant intravascular volume loss, reduced tissue perfusion, ischemia of the pancreas, and, in severe cases, necrosis, culminating in shock and potential multi-organ dysfunction. Although most AP episodes are mild, approximately 10-20% can progress to severe forms marked by sustained organ failure and pancreatic necrosis. AP often triggers a systemic inflammatory response syndrome (SIRS), which can be either short-lived or persist beyond 48 hours [23]. The negative impact of LC on the progression and outcomes of AP is likely compounded by a range of coexisting medical conditions frequently seen in these patients, such as diabetes mellitus, hypertension, obesity, cardiovascular disorders, chronic kidney disease, and chronic respiratory illnesses [24]. 

It remains uncertain if the effect of LC is observed independently of the confounders. We prioritize our analysis using adjusted models. If not available, then we used unadjusted models. However, due to the scarcity of studies assessing LC impacts on clinical outcomes in AP, it became impossible to perform an analysis of several important outcomes, like local and systemic complications. We performed subgroup analysis on adjusted and unadjusted estimates separately for all-cause mortality, and we did not observe any difference in estimates between the two subgroups. However, due to a smaller number of studies, more multicenter studies are required to validate these findings. 

High heterogeneity was observed in our meta-analyses for all-cause mortality and AKI. For AKI, we did not conduct subgroup analysis due to the limited number of included studies. However, for all-cause mortality, we performed subgroup analysis which confirmed the findings of our primary pooled analysis, reinforcing the consistency of the relationship between LC and increased mortality in AP. 

Our analysis provides critical insights by demonstrating that individuals with LC who develop AP experience significantly poorer hospital outcomes, including elevated mortality, compared to those without cirrhosis. These findings emphasize the importance of early identification of high-risk patients and the implementation of vigilant clinical monitoring. Given the complexity of managing AP in the setting of liver dysfunction, a coordinated, multidisciplinary treatment approach is essential. Involvement of hepatologists for cirrhosis management, nephrologists when renal impairment is suspected, and infectious disease specialists when infection is a concern may collectively enhance clinical outcomes. Prompt, collaborative care strategies can substantially improve the prognosis of cirrhotic patients hospitalized with AP. 

This meta-analysis is subject to several constraints. Primarily, all included studies were retrospective in design, inherently limiting the strength and reliability of the evidence. Additionally, the available literature lacked sufficient granularity to enable comprehensive subgroup analyses based on distinct causes or fibrosis stages of liver disease. Furthermore, important clinical endpoints such as AKI and disease severity were frequently unreported or inconsistently assessed across studies. Despite these limitations, the findings underscore the need for prospective, well-designed studies to validate and expand upon the current evidence. Future research should aim to explore more nuanced subgroups and consistently report key clinical outcomes to better inform risk stratification and personalized management strategies in patients with liver disease. 

## Conclusions

This meta-analysis demonstrates that LC significantly impacts outcomes in patients with AP, resulting in higher mortality (RR: 2.09) and increased disease severity (RR: 2.79). The consistent findings across methodologically robust studies with adjusted estimates further validate these associations. While no significant difference was observed in AKI risk, substantial heterogeneity warrants cautious interpretation. The coexistence of cirrhosis and AP poses unique management challenges due to altered inflammatory responses, impaired hepatic clearance, and compromised hemodynamics. These findings emphasize the importance of early risk stratification and multidisciplinary approaches involving hepatology, nephrology, and critical care specialists to treat this vulnerable population. Future prospective studies should address current limitations by examining cirrhosis etiology, severity stratification, and specific organ failures to further refine personalized management strategies and improve outcomes in this high-risk patient group.
